# Ophthalmology

**Published:** 1912-09

**Authors:** A. Ogilvy


### OPHTHALMOLOGY.

The subject of **miner's nystagmus** has been very much to the fore of late. Now that it has been made one of the scheduled industrial diseases, the colliery proprietors as well as the Government, have been making special inquiries and investigations into its cause and cure, if there is such a thing as a cure. Here in Bristol one does not see quite so many cases as formerly, although cases are not infrequent. A very interesting discussion took place in July at the Oxford Ophthalmological Congress. All the leading ophthalmic men from the colliery districts in South Wales were present, as well as many others from the North and Midland Counties of England, where collieries abound. One was rather astonished to hear of the frequency of the trouble. Several men who have the opportunity of seeing the collier at his work, and coming out of the mine after eight hours of semi-darkness, declared that it was as high as 50 per cent. Some even went as high as 75 per cent. The late Simeon Snell, of Sheffield, was a great advocate of the cause being the faulty position in which the collier worked, while the late Tatham Thompson, of Cardiff, was equally strong in maintaining that position had nothing to do with it, and that it was caused entirely by the work being done in semi-darkness. The discussion was long and slightly acrimonious, as both the disputants were strong fighters. Eventually the Government accepted the theory upheld by Snell, and this is the theory at present officially held by the Board of Trade. To show how opinions alter, it may be mentioned that when three or four members had spoken advocating "the want of light" theory of the cause, the chairman said, "To broaden the discussion a little, we should now like to hear someone who believes in the position theory." Out of thirty-five ophthalmic surgeons and four specially-appointed medical inspectors, not one was found to advocate the position theory. This position theory was founded on the idea that in cutting coal the collier lay on his right or left side, and looked upwards and outwards towards the point of his pick, thus bringing into play his oblique muscles. To prove his point, Snell maintained that he had seen a case in an ordinary chimney-sweep. One knows that this is the position of the eyes during that necessary aid to our domestic life. Now that inspectors have gone down mines to see the men at work, it is admitted that the collier never works above the level of the eyes, but always below. It is also admitted that in mines where the naked light is used miner's nystagmus is unknown. It was pointed out by several that in a great many cases of nystagmus there was a faulty focus of the eyes, which might be looked on as a contributory cause. Several others insisted that the nystagmus was only one symptom in a disease which they called miner's neurosis, and which is characterised by spasm of the orbicularis, photophobia, blinking and loss of vision. The importance of the last symptom was that the miner failed to see the small blue cone of light which forms over the safety lamp in gassy mines, and acts as a warning that the ventilation is not right before any actual disaster occurs. Unfortunately, the time for the discussion was limited to three hours, and the chairman had to decide to rule out the faulty focus and neurosis part, and confine members to nystagmus *per se.* The upshot of the discussion was the adoption of a resolution, carried unanimously, and to be forwarded to the Board of Trade, to the effect that the cause of miner's nystagmus was insufficient lighting, and that the position theory must be given up.

At the same Congress a useful paper was read by Professor Straub, of Amsterdam, entitled **Scrofulosis,** which is very much the same as struma, and is known as the cause of many ailments of eyes, which one hesitates to call tubercular, but which are no doubt connected with tubercle, though the tubercle bacillus is not actually found in the lesions. His theory is that the tubercle bacilli, having entered the body, may accumulate in the retroperitonal or other collections of glands, that there they remain for some time, producing no symptoms, and during that time they are altered and their virus attenuated. Eventually they may break out and attack various organs, causing a modified tubercular disease. If this explanation is true—and it sounds plausible—it will be a great comfort to many people who at meetings of medical societies describe a condition as being "strumous," and are at once questioned by the ultra-pathologist as to what he means by the word strumous.

As to the treatment of **syphilitic eye affections,** a paper was read and a discussion took place at the Ophthalmic Section of the British Medical Association in July on the use of salvarsan. Mr. Sydney Stephenson opened the discussion. He was strongly of opinion that salvarsan was most useful in irido-cyclitis coming on during a syphilitic attack, and he had never found any trouble with the optic nerve due to its use. He suggested that mercury should be used during the treatment, and in this he was strongly backed up by most of the speakers. It was generally agreed that while salvarsan acted very well in the treatment of iritis, it was practically useless in interstitial keratitis, which is of course also a syphilitic affection in 75 per cent. of the cases. One great difference, however, is that while iritis occurs in the acquired disease, interstitial keratitis is generally found in the inherited form, although it sometimes appears during the course of an acquired case. It has been said that owing to the small amount of blood which enters the eye, these specific remedies have not as much effect as they have in lesions in the rest of the body.

At the same meeting a discussion took place on **irido-cyclitis,** not of the specific variety. The discussion was opened by Mr. George Coats. As time goes on, and the cause of iritis is more and more ventilated, the old theory that iritis is caused by rheumatism and gout is dying out. Mr. Ormond mentioned that of 1,963 cases of acute rheumatism treated at Guy's Hospital, there were so few iritis cases that this cause of the disease must be given up. It was generally admitted that irido-cyclitis—as iritis alone seldom, if ever, occurs—was the result of general septic intoxication, usually from the intestinal tract. Many cases were known to result from pyorrhœa alveolaris, and disappeared when the pyorrhœa was cured. And so one by one all our old theories of eye diseases are disappearing, and before long an entirely new ophthalmology will have to be written.

A. OGILVY.

